# Natriuretic peptides modify *Pseudomonas fluorescens *cytotoxicity by regulating cyclic nucleotides and modifying LPS structure

**DOI:** 10.1186/1471-2180-8-114

**Published:** 2008-07-09

**Authors:** Wilfried Veron, Nicole Orange, Marc GJ Feuilloley, Olivier Lesouhaitier

**Affiliations:** 1 Laboratory of Cold Microbiology, UPRES EA 2123, University of Rouen, 55 rue Saint Germain, 27000 Evreux, France

## Abstract

**Background:**

Nervous tissues express various communication molecules including natriuretic peptides, *i.e*. Brain Natriuretic Peptide (BNP) and C-type Natriuretic Peptide (CNP). These molecules share structural similarities with cyclic antibacterial peptides. CNP and to a lesser extent BNP can modify the cytotoxicity of the opportunistic pathogen *Pseudomonas aeruginosa*. The psychrotrophic environmental species *Pseudomonas fluorescens *also binds to and kills neurons and glial cells, cell types that both produce natriuretic peptides. In the present study, we investigated the sensitivity of *Pseudomonas fluorescens *to natriuretic peptides and evaluated the distribution and variability of putative natriuretic peptide-dependent sensor systems in the *Pseudomonas *genus.

**Results:**

Neither BNP nor CNP modified *P. fluorescens *MF37 growth or cultivability. However, pre-treatment of *P. fluorescens *MF37 with BNP or CNP provoked a decrease of the apoptotic effect of the bacterium on glial cells and an increase of its necrotic activity. By homology with eukaryotes, where natriuretic peptides act through receptors coupled to cyclases, we observed that cell-permeable stable analogues of cyclic AMP (dbcAMP) and cyclic GMP (8BcGMP) mimicked the effect of BNP and CNP on bacteria. Intra-bacterial concentrations of cAMP and cGMP were measured to study the involvement of bacterial cyclases in the regulation of *P. fluorescens *cytotoxicity by BNP or CNP. BNP provoked an increase (+49%) of the cAMP concentration in *P. fluorescens*, and CNP increased the intra-bacterial concentrations of cGMP (+136%). The effect of BNP and CNP on the virulence of *P. fluorescens *was independent of the potential of the bacteria to bind to glial cells. Conversely, LPS extracted from MF37 pre-treated with dbcAMP showed a higher necrotic activity than the LPS from untreated or 8BcGMP-pre-treated bacteria. Capillary electrophoresis analysis suggests that these different effects of the LPS may be due, at least in part, to variations in the structure of the macromolecule.

**Conclusion:**

These observations support the hypothesis that *P. fluorescens *responds to natriuretic peptides through a putative sensor system coupled to a cyclase that could interfere with LPS synthesis and thereby modify the overall virulence of the micro-organism.

## Background

By the virtue of the size of their genome and the abundance of regulatory genes, bacteria of the genus *Pseudomonas *can adapt to a multitude of environmental niches [1]. In addition, they express natural resistance to β-lactamins and several disinfectants, and consequently the opportunistic behaviour of *Pseudomonas *is problematic in hospitals [2]. *Pseudomonas aeruginosa *is one of the micro-organisms most commonly responsible for nosocomial diseases and other fluorescent *Pseudomonas *also cause human infections [3,4]. Some psychrotrophic species, such as *Pseudomonas fluorescens*, which are pathogens in cold blood vertebrates [5], are also cytotoxic to mammalian cells [6] and can generate virulent clinical strains able to grow at 37°C [4]. *P. fluorescens *and *P. aeruginosa *possess a specific affinity for neurons and glial cells and their binding to the target cells is associated with apoptosis and necrosis [6]. The action of *Pseudomonas *on nerve cells appears to be very specific [7] and mostly mediated by the lipopolysaccharide (LPS) [8-10].

In the host, and particularly in the vicinity of nerve cells, bacteria are exposed to multitude of information molecules (including neurotransmitters and neurohormones). The sensing of these eukaryotic signals may modulate the physiology of the bacteria and may potentially modify their cytotoxicity. Indeed, the physiology and virulence of bacteria can be modulated by diverse small signal molecules such as norepinephrine [11], epinephrine [12], neuropeptides including somatostatin [13], melanocortin peptides [14], immune modulators such as dynorphin [15] and interferon-γ [16] and natriuretic peptides, namely the brain natriuretic peptide (BNP) and the C-type natriuretic peptide (CNP) [10]. We demonstrated recently the existence of a putative natriuretic peptide sensor in *P. aeruginosa *PAO1 [10]. Natriuretic peptides may act in this bacterium through activation of a cyclase leading to an increase in the intra-bacterial cAMP concentration and to stimulation of the Vfr global regulator. The resulting modulation of the cytotoxicity correlates with major changes in the structure of the LPS. Biochemical studies revealed that LPS extracted from *P. fluorescens *generally causes apoptosis whereas the LPS purified from *P. aeruginosa *is a potent pro-necrosis factor [8]. These findings and studies of the thermoregulation of virulence [17] suggest a dissociation of the apoptotic and necrotic effects of the endotoxins in the two species. *P. aeruginosa *and *P. fluorescens *are closely related, but there has been no investigation of whether *P. fluorescens *carries a natriuretic peptide sensor system. The results such an investigation would provide information about the probability of expression of similar systems in the group of fluorescent *Pseudomonas*, and about interspecies factors of variability.

Here, we report a study of the effects of BNP and CNP on the growth, adhesion potential and virulence of *P. fluorescens in vitro *using a model of primary cultures of glial cells. The variations of bacterial cytotoxicity induced by BNP and CNP were reproduced using cell permeable stable analogs of cyclic nucleotides, molecules, naturally produced in eukaryotes upon coupling of the natriuretic peptides to their receptors. We studied changes in intra-bacterial concentrations of cAMP and cGMP after exposure of the micro-organisms to BNP and CNP. We also investigated the effects of stable analogs of the cyclic nucleotides on the cytotoxicity and chemical properties of bacterial LPS.

## Results

In our experimental conditions at 28°C, *Pseudomonas fluorescens *MF37 multiplied rapidly, following an exponential growth curve until reaching stationary phase 6 hours after the beginning of the experiment. Addition of BNP or CNP (10^-6 ^M) at the onset of the incubation or at the beginning of the stationary phase did not modify the general profile of the growth curve or the mean time required for doubling of the bacterial population. Similarly, the cultivability and colony forming potential of *P. fluorescens *MF37 on solid substrate was unchanged by exposure of the bacteria to BNP or CNP (10^-6 ^M) (data not shown).

### Effect of pre-treatment with natriuretic peptides on the potential of *Pseudomonas fluorescens *MF37 to provoke apoptosis in glial cells

**Figure 1:**
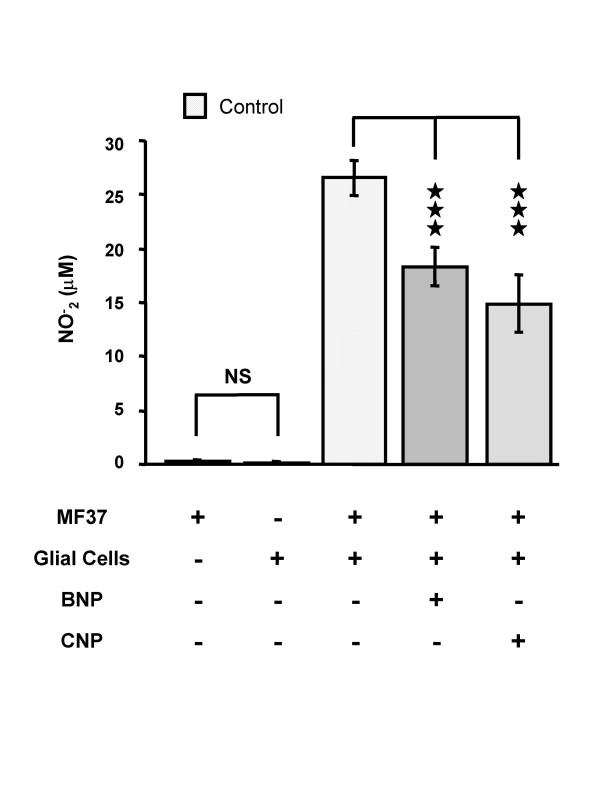
**Effect of brain natriuretic peptide (BNP) and C-type natriuretic peptide (CNP) (10^-6 ^M) on the apoptotic activity of *Pseudomonas fluorescens *MF37**. The apoptotic effect of the bacterium was determined by measurement of the accumulation in the medium of NO_2_^- ^resulting from the activation of inducible NO synthase activity in glial cells. Values are expressed as the mean concentration of NO_2_^- ^produced by glial cells following exposure (24 h) to untreated (n = 28) or treated (n = 24 for BNP, and n = 15 for CNP) bacteria. Data are means of four independent experiments. *** : Significantly different (*P *< 0.001). NS: Not significantly different.

The spontaneous nitrite (NO_2_^-^) synthase activity of *Pseudomonas fluorescens *MF37 is very low (Fig. 1); consequently, as previously demonstrated [17], NO_2_^- ^ions detected in the medium of cultures of glial cells exposed to *P. fluorescens *MF37 result from eukaryotic NO synthases activated during the induction of the apoptotic death of the glial cells. The concentration of NO^- ^_2 _in the medium following incubation of glial cells with *P. fluorescens *MF37 reached 26.5 ± 1.6 μM (n = 28). Pre-treatment of *P. fluorescens *with natriuretic peptides (10^-6 ^M for 5 h at 28°C) significantly decreased their capacity to provoke NO synthesis by glial cells: the NO^- ^_2 _concentration for bacteria exposed to BNP was 18.3 ± 1.8 μM (31 % lower than the control value; *P *< 0.001) and for those exposed to CNP was 14.9 ± 2.7 μM (46 % lower than the control value; *P *< 0.001) (Fig. 1).

### Effect of pre-treatment of *Pseudomonas fluorescens *MF37 by natriuretic peptides on its potential to provoke necrosis in glial cells

**Figure 2:**
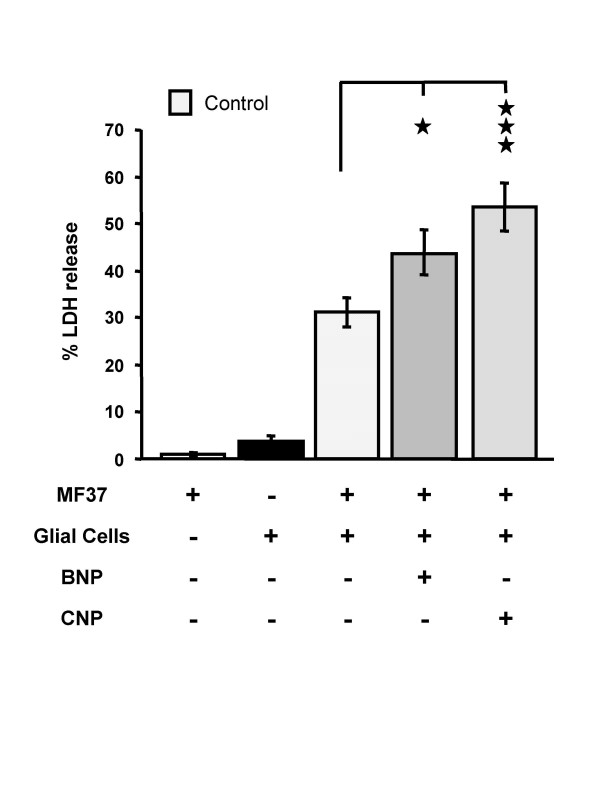
**Effect of brain natriuretic peptide (BNP) and C-type natriuretic peptide (CNP) (10^-6 ^M) on the necrotic activity of *Pseudomonas fluorescens *MF37**. The necrotic effect of the bacterium was determined by measurement of the accumulation in the medium of lactate dehydrogenase (LDH) resulting from rupture of the cytoplasmic membrane of glial cells and consequently release of the enzyme. Values are expressed as the mean concentration of LDH in the culture medium after 24 h of incubation with untreated (n = 20) or treated (n = 20 for BNP, and n = 17 for CNP) bacteria. Data are means for four independent experiments. * : Significantly different (*P *< 0.05). *** : Significantly different (*P *< 0.001).

Lactate dehydrogenase (LDH) is a stable cytosolic enzyme in eukaryotic cells and is totally absent from the medium when *Pseudomonas fluorescens *MF37 is incubated alone (Fig. 2); it can thus serve as a marker of eukaryotic cell lysis. When glial cells were incubated alone a small amount of LDH was recovered in the medium consistent with only a small proportion of the population (4.5 ± 1.0 %; n = 20) undergoing necrosis during the incubation period of 24 h. This indicates the good health and quality of the glial cell cultures. Glial cells were then exposed to *P. fluorescens *MF37 (10^6 ^CFU/ml): the percentage of the cell population affected by necrosis reached 31.2 ± 3.4 %. Pre-treatment of *P. fluorescens *with BNP or CNP (10^-6 ^M; 5 h; 28°C) resulted in a significant increase in the number glial cells affected by necrosis: 43.6 ± 4.8 % for bacteria exposed to BNP (40 % more than for untreated bacteria; *P *< 0.05) (Fig. 2) and 53.2 ± 5.1 % for bacteria treated with CNP (71 % more than for untreated bacteria; *P *< 0.001) (Fig. 2).

### Effect of pre-treatment of *Pseudomonas fluorescens *MF37 by stable analogues of cAMP and cGMP on its potential to provoke apoptosis and necrosis

**Figure 3:**
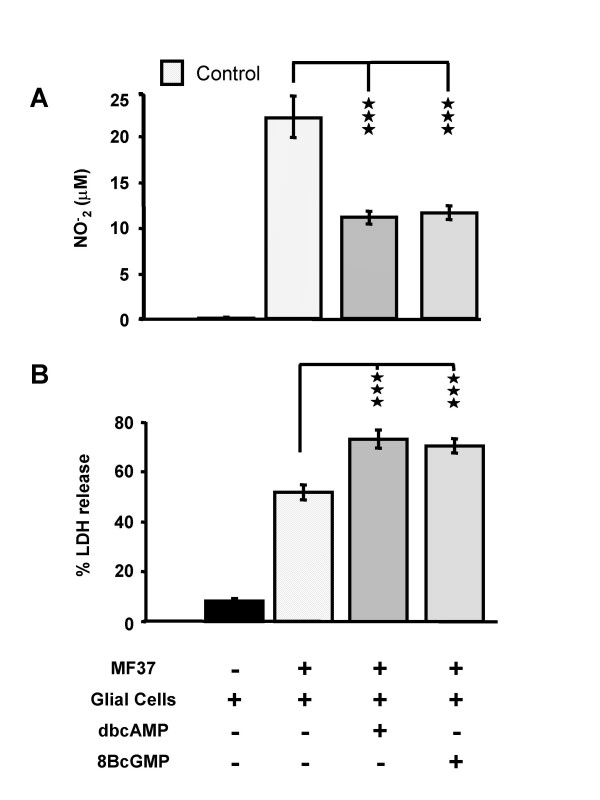
**Cytotoxic activities of *P. fluorescens *MF37 treated with cyclic nucleotide analogues**. Effect of dibutyryl cyclic AMP (dbcAMP) and 8-bromo cyclic GMP (8BcGMP) (10^-5 ^M) on the apoptotic (A) and necrotic (B) activities of *Pseudomonas fluorescens *MF37. The apoptotic effect of the bacterium was determined by measuring the accumulation of NO_2_^- ^in the medium resulting from the activation of inducible NO synthase activity in glial cells. The necrotic activity of the bacterium was assessed by measuring the accumulation of lactate dehydrogenase (LDH) in the medium resulting from rupture of the cytoplasmic membrane of glial cells. Values are expressed as mean concentrations of NO_2_^- ^or LDH in the culture medium after 24 h of incubation with untreated (n = 32) or treated (n = 24 for dbcAMP, and n = 22 for 8BcGMP) bacteria. Data are means for four independent experiments. *** : Significantly different (*P *< 0.001).

The cytotoxicity of *P. fluorescens *was investigated after exposure of the bacteria to dibutyryl cyclic AMP (dbcAMP) or 8-bromo-cyclic GMP (8BcGMP) (10^-5 ^M for 5 h at 28°C), two cell-permeable stable analogues of cyclic AMP and cyclic GMP. As observed using natriuretic peptides, pre-treatment of *P. fluorescens *with dbcAMP or 8BcGMP (10^-5 ^M for 5 h at 28°C) significantly decreased their capacity to provoke NO synthesis by glial cells: the NO^- ^_2 _concentration for bacteria exposed to dbcAMP was 11.2 ± 0.7 μM (50 % lower than the control value; *P *< 0.001) and for those exposed to 8BcGMP was 11.6 ± 0.8 μM (48 % lower than the control value; *P *< 0.001) (Fig. 3A). In contrast, a similar pre-treatment with dbcAMP or 8BcGMP increased the necrotic potential of the bacteria by +42.1 ± 5.1 % and +36.8 ± 4.1 %, respectively, of the control value (*P *< 0.001) (Fig. 3B).

Pre-treatment of *P. fluorescens *MF37 with BNP, CNP (10^-6 ^M for 16 hours), dbcAMP or 8BcGMP (10^-5 ^M for 16 hours) did not significantly modify the binding of bacteria to glial cells (data not shown).

### Effect of BNP and CNP on intra-bacterial cyclic AMP and cyclic GMP concentrations

**Figure 4:**
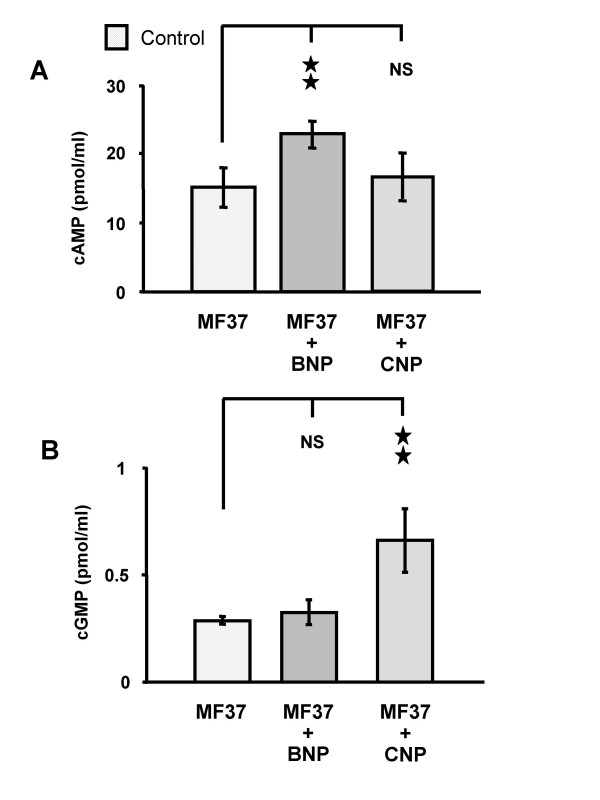
**Effect of natriuretic peptides on intra-bacterial concentration of monophosphate cyclic nucleotides**. Effect of brain natriuretic peptide (BNP) and C-type natriuretic peptide (CNP) (10^-6 ^M) on the intra-bacterial concentration of cyclic AMP (cAMP) (A) and cGMP (B) in *Pseudomonas fluorescens *MF37. Data are means for three independent experiments (n = 9) ** : Significantly different (*P *< 0.01). NS: Not significantly different.

The concentration of cyclic AMP (cAMP) in *P. fluorescens *MF37 in late exponential phase was 15.4 ± 3.0 pmol/ml. When *P. fluorescens *was incubated with BNP (10^-6 ^M, 30 min) the concentration of cAMP increased to 22.9 ± 2.1 pmol/ml (48.7 % above the basal level; *P *< 0.01) (Fig. 4A). CNP (10^-6 ^M, 30 min) had no significant effect (*P *> 0.05) on the intra-bacterial concentration of cAMP in *P. fluorescens*. The concentration of cyclic GMP (cGMP) in *P. fluorescens *MF37 was 0.28 ± 0.02 pmol/ml, and thus lower than that of cAMP; furthermore, this value was in the same range as the sensitivity of the assay. BNP (10^-6 ^M, 30 min) did not significantly modify the cGMP concentration in *P. fluorescens*, whereas CNP (10^-6 ^M, 30 min) caused an increase to 0.66 ± 0.15 pmol/ml of cGMP (136 % higher than the basal level; *P *< 0.01) (Fig. 4B).

### Effects of stable analogues of cAMP and cGMP on the cytotoxicity of the lipopolysaccharide of *Pseudomonas fluorescens *MF37

**Figure 5:**
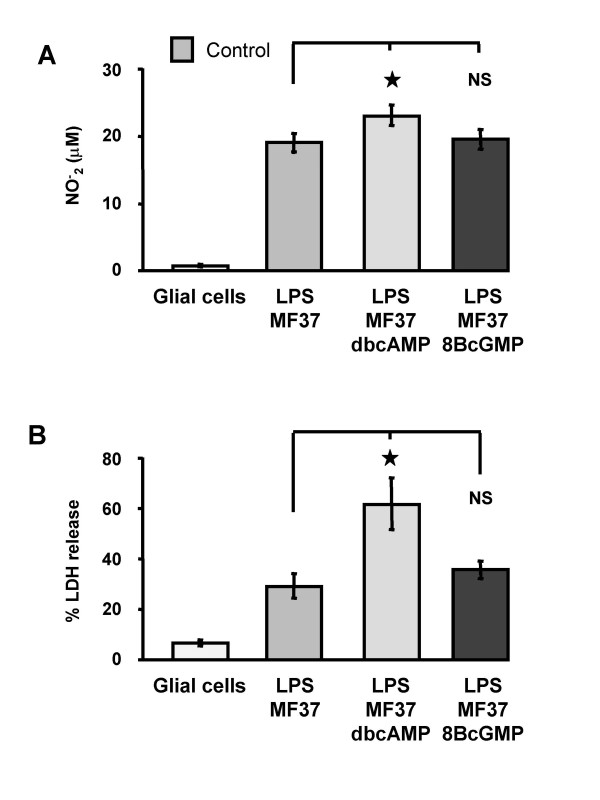
**Cytotoxic activity of LPS from *Pseudomonas fluorescens *MF37 treated with cyclic nucleotide analogs**. Effect of treatment of *P. fluorescens *MF37 with dibutyryl cyclic AMP (dbcAMP) and 8-bromo cyclic GMP (8BcGMP) on the apoptotic (A) and necrotic (B) activity of its LPS. The apoptotic effect of the LPS was determined by measurement of the accumulation of NO_2_^- ^in the medium resulting from the activation of inducible NO synthase activity in glial cells. The necrotic action of the LPS was assessed by measurement of the accumulation of lactate dehydrogenase (LDH) in the medium resulting from rupture of the cytoplasmic membrane of glial cells. Values are expressed as mean concentrations of NO_2_^- ^or LDH in the culture medium after 24 h of incubation with untreated (n = 9) or treated (n = 11 for dbcAMP, and n = 11 for 8BcGMP) LPS (500 ng.ml^-1^). Data are means for three independent experiments. * : Significantly different (*P *< 0.05). NS: Not significantly different.

LPS makes a large contribution to the cytotoxicity of *Pseudomonas *on glial cells [8]. We therefore evaluated the effects of stable analogues of cyclic nucleotides on LPS activity. The cytotoxicity of the LPS from *P. fluorescens *exposed to dibutyryl cyclic AMP (dbcAMP) or 8-bromo-cyclic GMP (8BcGMP) (10^-5 ^M for 4 h at 28°C) was compared to that of the LPS from control bacteria taken at the same stage of growth (early stationary phase). LPS from control and pre-treated bacteria were extracted and their concentrations were determined by a KDO assay. The mean concentrations of LPS samples from bacterial cultures grown in the absence (3.3 μg/ml) or presence of cAMP or cGMP (2.5 μg/ml and 4.1 μg/ml, respectively) were all in the same range. Equivalent aliquots were added to the culture medium (to give a final concentration of 500 ng/ml) of glial cells and cytotoxicity was determined, as previously described, by measurement of the concentration of NO^- ^_2 _(Fig. 5A) and by measurement of LDH release (Fig. 5B). When glial cells were exposed to LPS extracted from control *P. fluorescens *MF37, the NO^- ^_2 _concentration was 19.2 ± 1.4 μM (n = 9); the value obtained for LPS from *P. fluorescens *exposed to dbcAMP was significantly higher (*P *< 0.05) at 23.3 ± 1.5 μM (n = 11) (Fig. 5A) whereas that for LPS from *P. fluorescens *exposed to 8BcGMP was not significantly different to the value for LPS from control MF37 (Fig. 5A). Necrosis in glial cell cultures, in this series of experiments, affected 6.4 ± 1.1 % of the control cell population in 24 h (n = 8) (Fig. 5B). For glial cells exposed to LPS extracted from control *P. fluorescens *MF37, 29.2 ± 4.9 % of the cells displayed signs of necrosis. For glial cells treated with LPS from MF37 exposed to dbcAMP, 61.7 ± 10.4 % (*P *< 0.05) of the cell population was affected by necrosis (Fig. 5B). In contrast, LPS from MF37 exposed to 8BcGMP did not cause significantly more necrosis than control LPS (35.4 ± 3.6 % of the cell population affected, *P *> 0.05). Control tests with extracts of nutrient broth medium (ONB) obtained using the same LPS extraction and purification protocols indicated that the effect of the LPS on glial cell viability was not due to contaminants from the extraction buffers employed for purification (data not shown).

### Effect of stable analogues of cAMP and cGMP on the structure of the lipopolysaccharide of *Pseudomonas fluorescens *MF37

**Figure 6:**
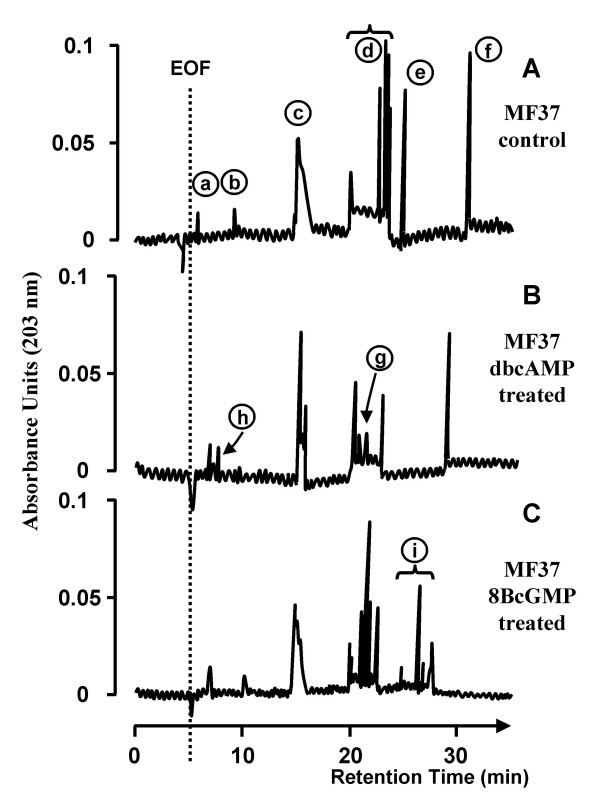
**MEKC analysis of LPS from *Pseudomonas fluorescens *MF37 treated with cyclic nucleotide analogs**. MEKC analysis of LPS extracted from control (A), dbcAMP (10^-5 ^M)-treated (B) and 8BcGMP (10^-5 ^M)-treated (C) *P. fluorescens *MF37. EOF, electo-osmotic flux. Arrows and numbers refer to the different molecular forms identified in the LPS from *P. fluorescens *MF37.

LPS extracted and purified from *P. fluorescens *MF37 was analysed by micellar electrokinetic chromatography (MEKC), a technique that allows the separation of non-volatile macro-molecules showing small differences of size and polarity with an efficiency in the same range as gas chromatography [18]. The LPS of *P. fluorescens *MF37 grown in control conditions gave numerous peaks (a to f, Fig. 6A). Peaks a and b were minor peaks with retention times of 7.0 and 11.1 min, respectively, each apparently corresponding to a single molecular form. Peak c was larger, with a retention time of 18.0 min and corresponding to a series of compounds with very closely related structures. Peak d was broad, with a retention time between 21.3 and 24.6 min and presented a large variety of molecular forms (Fig. 6A). Peaks e and f were high and thin, with retention times of 26.3 and 32.2 min, respectively. The electropherograms of LPS extracted from *P. fluorescens *MF37 treated with dbcAMP and 8BcGMP (10^-5 ^M for 4 h at 28°C) presented marked differences, although the same major peaks (c, d and f) were detected in all electropherograms (Fig. 6B and 6C). The major difference between LPS extracted from control and dbcAMP-treated bacteria was the smaller number of molecular forms after dbcAMP treatment (Fig. 6B): the broad spectrum of compounds (peak d) was substantially smaller whereas a new peak (g) appeared (Fig. 6B); the composition of compounds corresponding to the broad peak (c) appeared to be different and a new peak (h) appeared, corresponding to a weakly retained molecular form (Fig. 6B). The structure of the LPS extracted from 8BcGMP-treated bacteria was also different: a broad signal (i) corresponding to numerous new strongly retained compounds was found (Fig. 6C); in contrast as observed with dbcAMP-treated LPS, a compound eluting as peak b was detected.

## Discussion

We first tested for any direct antibacterial effect of the natriuretic peptides BNP and CNP on *Pseudomonas fluorescens*. The concentration of the natriuretic peptides was that usually employed to investigate the physiological activity of these molecules in eukaryotic cells [19] and identical to that previously tested on *P. aeruginosa *[10]. Because the cultivation method can greatly influence the survival of bacteria [20], we decided to distinguish between possible effects of BNP and CNP on growth on liquid and solid substrates. Bacterial growth curves and counting demonstrated that BNP and CNP had no effect on the growth or cultivability of *P. fluorescens *in our experimental conditions. This finding diverges from those of previous studies showing an antimicrobial activity of BNP [21] and suggesting a similar effect of CNP [22]. However, the previously described activity of BNP appeared essentially targeted towards Gram-positive bacteria; the minimum inhibitory concentration of BNP for *Pseudomonas *was 40 times that employed in our experiments [21]. Other than the structural homologies with anti-microbial peptides, the only reason for suspecting that CNP expresses bactericidal activity is that it can form channels in artificial lipid bilayers [22]. However, artificial membranes made of pure phospholipids are very different to the double and LPS-decorated membranes of Gram-negative bacteria. Moreover, in physiological conditions CNP is probably not present in sufficient concentrations to act as an ionophore.

As these natriuretic peptides were without effect on the survival of *P. fluorescens*, we used a well-characterised model of glial cell infection [6,8] to investigate the effect of BNP and CNP on the virulence of the bacteria. *P. fluorescens *was incubated with one or other natriuretic peptide during exponential growth, rinsed and then added to primary cultures of glial cells. Thus, eukaryotic cells were never exposed to the peptides and the effects observed can only result from changes in the virulence of the bacteria. We first showed that treatment of *P*. *fluorescens *by BNP and CNP decreased its apoptotic effect on glial cells. This suggests that the natriuretic peptides altered the bacterial physiology sufficiently to modify the production of virulence factors without affecting growth and survival. This is in agreement with studies showing that anti-microbial substances can exert other effects on bacterial physiology or on toxins without inhibiting the survival of the micro-organism [23,24]. Also, as described for *P. aeruginosa *[10], BNP and CNP treatment of *P. fluorescens *increased its necrotic activity. Necrosis is due to disruption of the cytoplasmic membrane, generally due to enzymatic activities, whereas apoptosis is a process induced by the production of cell death messengers. We have previously shown that the two mechanisms are differently regulated by *P. fluorescens *[17]. As suggested by Jungas *et al*. [25] a rapid and strong induction of necrosis can mask other apoptotic processes. Indeed, in our experimental conditions, the increase in NOS activity appears only 10 hours after the initial contact between glial cells and bacteria, whereas LDH release was markedly increased within 4 hours (data not shown). This necrosis may be triggered by bacterial secretion of cytotoxic enzymes or virulence factors. In support of this hypothesis it was recently shown that MFN 1032, a clinical strain of *P. fluorescens *[4], secrets extracellular factors, including a phospholipase C, with lytic potential in the same range as that of *P. aeruginosa *[26]. Note that the increase of bacterial virulence due to treatment by natriuretic peptides was not associated with an increase in bacterial adhesion to the target cells and therefore does not appear to be contact mediated. Nevertheless, variations in the efficiency of toxin translocation through a type III secretion system would also explain our results; *P. aeruginosa *expresses a type III secretion system [27], but no such system in *P. fluorescens *MF37 has been described.

In order to go further into the mechanism involved in the modulation of *P. fluorescens *cytotoxicity by natriuretic peptides, we studied the effects of stable analogues of cyclic nucleotide monophosphate. We used cell-permeable stable analogues of cGMP and cAMP as tools to explore the action of BNP and CNP in *P. fluorescens*, in view of possible analogy with the mechanisms of action of natriuretic peptides in eukaryotes. Indeed, in these cells the effects of natriuretic peptides are mediated by three different receptor subtypes, NPR-A, NPR-B and/or NPR-C which are all associated with adenylate or guanylate cyclase activities [28,29]. We observed that exposure of *P. fluorescens *to 8-bromo-cyclic GMP (8BcGMP) and dibutyryl cyclic AMP (dbcAMP) fully reproduced the dual effect of BNP and CNP, *i.e*. a decrease of apoptosis and an increase of necrosis. The involvement of cAMP in the regulation of host-directed virulence factors, through the cAMP binding protein Vfr, has been demonstrated for *P. aeruginosa *[30], but the effects of cGMP were unknown until now. In *P. aeruginosa*, the Vfr protein may be activated by both cAMP and cGMP, as Vfr is unlikely to discriminate between the two types of cyclic nucleotide [31]. Note that a cyclic nucleotide recognition module, called "GAF" and present in a wide range of species from bacteria to human, has been described [32]. This module binds both cAMP and cGMP and it is possible that some cyclic nucleotide binding proteins in bacteria do not discriminate between the two types of cyclic nucleotide mono-phosphate. The involvement of a cyclic nucleotide-dependent cascade in the action of natriuretic peptides in *P. fluorescens *was confirmed by a direct assay of cAMP and cGMP: BNP provoked a significant rise in the cAMP concentration in the bacterial stroma but was without effect on cGMP. Conversely, CNP induced an increase of the intra-bacterial concentration of cGMP but not that of cAMP. These findings are noticeably different from those for *P. aeruginosa *in which BNP has no effect on the cAMP concentration [10]. Thus, it is suggesting that the sensitivity to natriurectic peptides diverges between *Pseudomonas *species. *P. fluorescens *and *P. aeruginosa *therefore have different responses to BNP and CNP and consequently it is likely that there are at least two forms of putative natriuretic-peptide sensor in *Pseudomonas *species. Consistent with this notion, the bacterium *Helicobacter pylori *can recognise only one of the subtypes of the somatostatin receptor agonists and thus displays highly stereospecific recognition of this eukaryotic messenger [13]. Here, we demonstrate for the first time, that cGMP, a ubiquitous eukaryotic second messenger, is involved in the regulation of an intra-bacterial transduction signal. This illustrates the remarkable analogy between the effects of natriuretic peptides in eukaryotes and prokaryotes. The number of adenylyl and guanylyl cyclases identified in bacteria is now substantial [33] and is consistent with our observations: bacterial cyclase(s) may be involved in the response of *P. fluorescens *to natriuretic peptides suggesting that in addition to the well-characterised bacterial second messenger, cyclic di-GMP [34], cyclic monophosphate nucleotides may play crucial roles in the integration of environmental signals transmitted from the bacterial surface.

The *Pseudomonas *endotoxin, LPS, is a major virulence factor released upon bacterial death, but can also be produced as vesicular forms by living bacteria [35,36]. The composition of the LPS produced by *P. fluorescens *can change rapidly under stress conditions [17]. We studied the effects of the stable and cell-permeable analogues of cyclic nucleotide monophosphate on the cytotoxic activities and chemical properties of the LPS. It would have been preferable to use the natriuretic peptides themselves but the cost was prohibitive in regard of their consumption by such experiments that require producing massive concentrations of LPS. Modifications of the LPS structure are directly associated with differences in its pro-necrotic activity [8,17], but did not affect the pro-apoptotic effect of the endotoxin (this study). Thus, endotoxin-induced cell death may be ascribed to at least two distinct processes. Bacterial-induced cell death is undoubtedly even more complex as it may involve not only apoptosis and necrosis, but also from the more recently described pyroptotic system [37]; furthermore, a single factor or micro-organism can simultaneously induce different death types [38]. This and previous works with *P. fluorescens *[8,17] suggest that the expression of the LPS components involved in the initiation of necrosis may be regulated through a cAMP-dependent intra-bacterial pathway, whereas pro-apoptotic factors are regulated by a different mechanism. As observed in *P. aeruginosa *[10], greater cytotoxicity of the LPS appeared to be associated with a decrease in the diversity of the LPS isoforms. Further investigations will be necessary to confirm if the lipid A part of the LPS from *P. fluorescens*, like its counterpart in *P. aeruginosa *PAO1 [10], is the target of the modification associated with the intra-bacterial cyclic nucleotide concentration.

## Conclusion

We demonstrate the existence of a natriuretic peptide sensor mechanism in *Pseudomonas fluorescens *and the involvement of cyclase(s) in the response to BNP and CNP. For the first time we report that cGMP, in addition to cyclic di-GMP and cAMP, may be involved in the coupling of the bacterial response to specific extracellular messengers. This work opens a wide range of possibilities for research concerning the reasons for preservation – or common emergence – of a natriuretic peptide-dependent receptor/sensor system during evolution in eukaryotes and prokaryotes. Studies of the physiological role of natriuretic peptides in bacteria will undoubtedly be informative. Our work also suggests that the number of sensor or communication molecules recognised by Gram-negative bacteria has been probably substantially underestimated.

## Methods

### Reagents and test substances

Dulbecco's Modified Eagle's Medium (DMEM) and Ham's F12 culture medium, HEPES buffer, poly-L-lysine, insulin, dibutyryl cyclic AMP (dbcAMP), 8-bromo-cyclic GMP (8BcGMP), and human BNP were purchased from Sigma-Aldrich (St Quentin Fallavier, France). CNP peptide was from NeoMPS (Strasbourg, France). Foetal calf serum, L-glutamine and antibiotic-antimycotic solutions were obtained from Biowhittaker (Emerainville, France). The kit Cytotox 96 was purchased from Promega (Charbonnières, France).

### Bacterial cultures

*Pseudomonas fluorescens *MF37 is a biovar V, naturally rifampicin-resistant mutant of the psychrotrophic strain MF0 selected in our laboratory [39]. The strain was grown in nutrient Broth n°2 medium (ONB) (AES, Bruz, France) at 28°C. For pre-treatment with natriuretic peptides or cyclic nucleotide analogues, bacteria were transferred to 10 ml ONB and the test substances were added at the beginning of the exponential phase; the cultures were then grown for 5 h. Just before the infection assays, bacteria in early stationary phase were harvested by centrifugation in an Eppendorf centrifuge tube (6000 rpm for 4 min at 20°C) and resuspended at a cell density of 10^6 ^CFU/ml, in glial cell culture medium without antibiotics or antimycotics. The density of the bacterial suspension was determined by measuring absorption at 580 nm using a spectrophotometer (ThermoSpectronics, Cambridge, UK). The bacterial density and the absence of contamination were verified by plating. The effects of natriuretic peptides on bacterial growth kinetics were studied at 28°C. The natriuretic peptides (BNP or CNP; 10^-6 ^M) were administered either immediately at the start of the experiment or at the beginning of the early stationary phase. To determine the effects of natriuretic peptides on bacterial cultivability, *P. fluorescens *MF37, taken at the beginning of the stationary phase, was incubated for 5 h with BNP or CNP (10^-6 ^M) and then plated.

### Primary cultures of glial cells

Newborn rats obtained by mating in the laboratory were decapitated 48 – 72 h after birth under sterile conditions. All animal manipulations were performed under the supervision of qualified investigators and in authorised animalery (Agreement n° AGEXP27.01). The brain was quickly extracted and rinsed in glial culture medium consisting of DMEM/Ham's medium (2 : 1) supplemented with 10 % foetal calf serum, 2 mM glutamine, 0.001 % insulin, 5 mM Hepes, 0.3 % glucose and 1 % antibiotic – antimycotic solution. The meninges were removed and the telencephalon was carefully dissected, immersed in glial culture medium and mechanically dispersed for 5 min by gentle aspiration through a sterile needle. The suspension was filtered through a sterile 82 μm pore-size nylon filter to remove the remaining tissue fragments. The cells were counted and 10^5 ^cells were layered in each 1.9 cm^2 ^well coated with poly-L-lysine 50 μg/ml. Glial cells were incubated at 37°C in a 5 % CO_2 _humidified atmosphere and were allowed to grow for 9 to 14 days, so as to obtain confluent cells in all culture wells. The culture medium was changed the first day after plating and then every 2 days.

The binding index (adhesive behaviour) of *P. fluorescens *MF37 on glial cells was investigated using the gentamicin assay adapted from that used to quantify *P. aeruginosa *invasion of epithelial cells [40].

### Measurement of the nitric oxide (NO) synthase activity of glial cell

NO synthase activity of glial cells is an indirect marker of apoptosis that has been previously validated by Annexin V and propidium iodide double staining observations using staurosporin as a positive control to visualise cytoskeletal rearrangement [8]. NO synthase activity was assayed by measuring the accumulation of nitrites (NO_2_^-^) in the culture medium using a technique derived from the Griess colorimetric reaction [41]. To allow the accumulation of nitrite in the medium, the assays were performed after 24 h of incubation of glial cells with control or pre-treated bacteria. The Griess reagent was prepared extemporaneously by mixing equal volumes of N-(1-naphtyl) ethylenediamine and sulphanilic acid. This solution was mixed (V/V) with 500 μl of cell culture supernatant filtered through a 0.22 μm pore-size filter. After incubation for 30 min at room temperature, the absorbance was measured at 548 nm. A standard curve was obtained by serial dilution of a solution of NaNO_2 _from 100 to 2 μm. The linearity of the assay between these concentrations was verified and the concentrations in the assay were kept between these values. The intra- and between-assay coefficients of variation were lower than 3 and 8 %, respectively.

### Measurement of the release of cytosolic lactate dehydrogenase (LDH) by glial cells

Lactate dehydrogenase (LDH) is a stable cytosolic enzyme released into the culture medium upon cell lysis. Its use as an indicator of necrosis in glial cells has been validated by epifluorescence observations using SDS as a positive control [8]. The amount of LDH released by eukaryotic cells in the presence of the bacteria was determined using the Cytotox 96^® ^enzymatic assay (Promega, Charbonnières, France). Glial cells were incubated for 24 h with control or pre-treated *P. fluorescens *MF37 at a concentration of 10^6 ^CFU/ml. A lysis buffer, consisting in a solution of Triton X-100 (9 % in water), was employed to determine the maximum of LDH possibly released by glial cells in our experimental conditions (100 % LDH release). A background level was established using culture medium alone, and defined as 0% LDH release, to eliminate the contribution of the culture medium. The percentage of LDH release in the cell population was then calculated using the equation:

$$\%\text{LDH}=\frac{\left(\text{OD sample}-\text{OD }0\%\right)}{\left(\text{OD 1}00\%\;-\text{OD }0\%\right)}\times 100$$

(OD = Optical Density)

The assay was sufficiently sensitive to measure a concentration of LDH equivalent to the lysis of 1 % of the cell population.

### Determination of intra-bacterial cyclic AMP and cyclic GMP

The concentrations of cyclic AMP (cAMP) and cyclic GMP (cGMP) in bacteria exposed to natriuretic peptides were determined using cAMP and cGMP direct enzyme immunoassay kits from Sigma RBI (Saint-Quentin Fallavier, France). Aliquots of *P. fluorescens *(10^6 ^CFU/ml) in late exponential phase were incubated for 30 min at 28°C in 10 ml ONB containing BNP or CNP (10^-6 ^M). The bacteria were then centrifuged (6 min at 6.000 rpm) and the pellets were resuspended in physiological water and boiled for 10 min. These extracts were ultra-centrifuged (1 h at 62 000 *g*) to remove cell debris and then lyophilised. Dried residues were resuspended in 1 ml of water and assayed for cAMP or cGMP according to the supplier's protocol.

#### Lipopolysaccharide extraction and purification

LPS was purified from *Pseudomonas fluorecens *MF37 according to a procedure based on that of Darveau and Hancock [42]. Briefly, bacteria in early stationary phase were harvested by centrifugation (6 000 × *g *for 10 min at 20°C). The pellets were suspended in 10 mM Tris-buffer containing 2 mM MgCl_2_, 200μg/ml pancreatic DNase and 50μg/ml pancreatic RNase, and subjected to four bursts of sonication of 30s at a probe density of 70. The suspension was then incubated for 2 h at 37°C and tetrasodium-EDTA, Tween 20 and tris-hydrochloride were added. The sample was centrifuged (10 000 × *g*, 30 min, 20°C) to remove peptidoglycan and the supernatant was incubated overnight with protease, at 37°C, with constant shaking. Two volumes of 0.375 M MgCl_2 _in 95% ethanol were added and the mixture was cooled to 0°C. The sample was then centrifuged (12 000 × *g*, 15 min, 0°C) and the pellet was sonicated in a solution of Tween 20 and tetrasodium-EDTA. The pH of the solution was lowered to 7, to prevent lipid saponification. The solution was incubated for 30 minutes at 85°C, to ensure that outer membrane proteins were denatured, and the pH of the solution was increased to 9.5. Protease was then added and the sample was incubated overnight at 37°C. Two volumes of 0.375 M MgCl_2 _in 95% ethanol were added and the sample was centrifuged (12 000 × *g *for 15 min at 0°C). The pellet was resuspended in 10 mM Tris-HCl, sonicated and centrifuged twice to remove insoluble Mg^2+^-EDTA crystals. The supernatant was then ultracentrifuged (62 000 × *g *for 2 h at 15°C) and the pellet, which contained LPS, was resuspended in distilled water and used for KDO assays. The control extraction buffer was prepared by the same procedure, but starting with sterile ONB medium.

#### Capillary electrophoresis of lipopolysaccharide

Purified bacterial LPS was analysed by micellar electrokinetic chromatography (MEKC), using a Beckman P/ACE 5510 system equipped with a diode-array detector (detection range: 190–600 nm, wavelength of the curves presented: λ = 203 nm) and refrigerated injection system. A fused-silica capillary tube (50 μm i.d. × 57 cm; 50 cm to detector) from Beckman Coulter (Villepinte, France) was used: it was installed in a refrigerated cartridge and conditioned with 1 N NaOH for 4 h before rinsing and equilibration with running buffer. The separation buffer for MEKC consisted of 25 mM boric acid (pH 9.00) supplemented with sodium dodecyl sulphate (80 mM). The potential used for separation (25 kV) was identified in preliminary experiments as the limit of linearity of the curve for the current-voltage relationships. Samples were injected by N_2 _hydrostatic pressure (0.5 psi, 20 s) and analysed over a 30-minute period.

### Statistics

Each value reported for the assays is the mean of measurements of a minimum of nine samples from a minimum of three independent preparations. The non parametric Mann-Whitney test was used to compare the means within the same set of experiments.

## Authors' contributions

WV participated in the experimental design and carried out most of the experimental studies. NO participated in designing the study. MGJF coordinated the study and wrote the manuscript. OL carried out a part of the measurement of intra-bacterial cyclic AMP and cyclic GMP, coordinated the study and contributed to drafting the manuscript. All authors read and approved the manuscript.
